# Acute Myeloid Leukemia in Infants: Biology and Treatment

**DOI:** 10.3389/fped.2015.00037

**Published:** 2015-04-28

**Authors:** Riccardo Masetti, Francesca Vendemini, Daniele Zama, Carlotta Biagi, Andrea Pession, Franco Locatelli

**Affiliations:** ^1^Hematology-Oncology Unit “Lalla Seràgnoli”, Department of Pediatrics, University of Bologna, Bologna, Italy; ^2^Department of Pediatric Hematology-Oncology, IRCCS Ospedale Bambino Gesù, University of Pavia, Pavia, Italy

**Keywords:** acute myeloid leukemia, infants, prognostic factors, toxicity, hematopoietic stem cell transplantation, treatment results

## Abstract

Children aged 0–2 years (i.e., infants) with acute myeloid leukemia (AML) are a peculiar subgroup of patients in the childhood AML scenario. They present with distinctive biological and clinical characteristics, including a high prevalence of prognostically unfavorable risk factors and an increased susceptibility to therapy-related toxicity. Remarkable improvements have been achieved over the last two decades in the treatment of these patients and their outcome is becoming superimposable to that of the older age groups. In this review, we will focus on peculiarities of this young subgroup of children with AML, describing their clinical presentation, the biology of disease, and factors influencing outcome. Treatment results and toxicity data reported by major collaborative groups are also summarized and compared.

## Introduction

Prognosis of childhood acute myeloid leukemia (AML) has improved significantly over the past decades, the current probability of cure being approximately 60% in most of the developed countries (1). This result has been achieved thanks to not only the use of more effective anti-leukemic agents and significant improvements in supportive care, but also to the progress made in the stratification of patients with a consequent risk-directed therapy (2, 3). Children <1 year of age (i.e., infants) with AML represent a peculiar subgroup of patients with distinctive clinical and biological features (1, 2). Infants with AML have been historically deemed to be high-risk (HR) patients, due to the high prevalence of prognostically unfavorable features (4, 5) and a greater vulnerability to treatment-related toxicity (3–5). Nevertheless, during the last two decades, many cooperative groups have reported an outcome of infants not different from that of older children (4, 6–8): in other words, the remarkable differences in terms of molecular lesions of infant AML (9) seem no longer to be associated with a dismal prognosis (6–8).

In this review, we provide an overview of the most relevant international studies focused on infants with AML discussing the clinical features, genetic characteristics, and treatment outcome. As a preliminary consideration, we emphasize that, over the years, many studies combined data of children <12 months of age with those of 1- to 2-year-old patients in a single group. Although historically, the conventional definition of infant AML includes children <1-year–old; in this review, we chose to focus on characteristics of children under 2 years of age, since children under 1 year and those aged between 1 and 2 years have similar features and superimposable survival results.

## Clinical and Biologic Characteristics of Infant AML

### Clinical features

Infants with AML present with clinical characteristics distinct from that of older children, including a higher incidence of acute monoblastic, myelomonoblastic, and megakaryoblastic leukemia [M4, M5, and M7 subtypes in the French-American-British (FAB) classification], higher leukocyte count at diagnosis, as well as higher frequency of central nervous system (CNS) and extramedullary involvement (2, 4). Indeed, nearly 70% of infants with AML have M4/M5 and M7 AML (50 and 20% for M4/M5 and M7, respectively) (4, 6–8), while FAB M1 and M2 are most common subtypes in older children (6, 8).

Young age has also been reported to be associated with the occurrence of extramedullary organ (usually skin) and CNS involvement at diagnosis, the latter being defined by the presence of 5 × 10^6^/L or more white blood cells (WBCs) in the cerebrospinal fluid (4, 6, 8). The incidence of CNS disease reported by major collaborative group spans from 12–24% in children aged <2 years to 3–7% in older children (4, 6, 8). The higher incidence of CNS disease may be due to the greater vasculature of infant leptomeninges (10) and to the prevalence of monoblastic leukemia in infants, since the leukemia counterpart of monocytes can retain the physiological ability to migrate into peripheral tissues and to reach the brain passing through the endothelial cell cytoplasm (4). Considering other extramedullary organ involvement, data are not univocal, since some studies documented a higher incidence in children aged <2 years in comparison with older patients (31–36 vs 21%, *P* = 0.0015 in one BFM report) (4), whereas no significant age-related differences have been reported in other studies [12–15 vs 17–18%, *P* = 0.8 in the Associazione Italiana di Ematologia e Oncologia Pediatrica (AIEOP) analysis] (8).

The same kind of discrepancy among different reports pertains to hyperleukocytosis (i.e., a WBC count greater than 100 × 10^9^/L) at diagnosis, since one study reported that infants with AML have a higher frequency of hyperleukocytosis (28% in patients aged <1 year vs 14% in children older than 2 years, *P* = 0.003) (4), while other groups did not confirm this finding (6–8).

### Cytogenetic abnormalities

The chromosomal aberrations of AML in children younger than 2 years are peculiar and markedly different from those found in older children (2).

**Figure 1:**
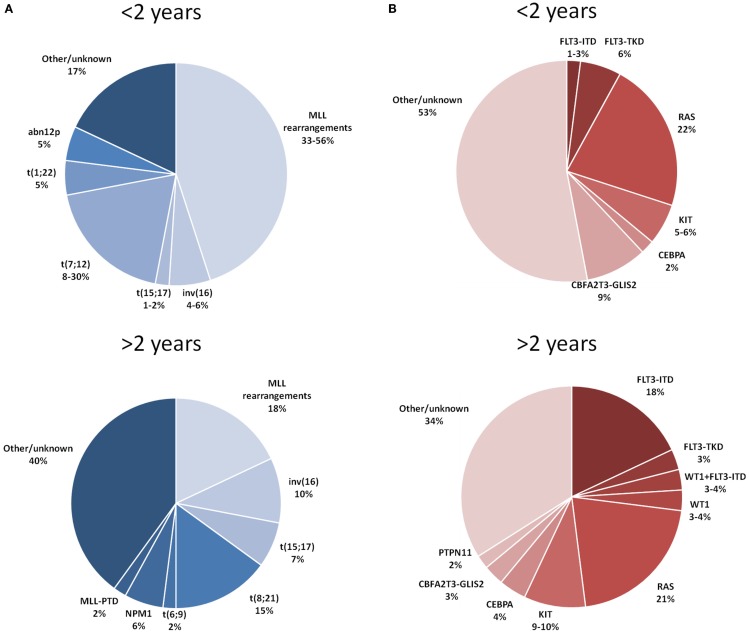
**Distribution of the main cytogenetic (A) and molecular (B) aberrations reported in children with AML younger and older than 2 years of age**. References: Balgobind et al. (9) and Masetti et al. (11).

Figure 1A depicts the distribution of the main recurrent cytogenetic abnormalities of AML in children younger and older than 2 years of age.

Translocations involving chromosome band 11q23, mostly in myelomonocytic or monoblastic subtypes, are the most common recurrent cytogenetic aberrations detected in infants (9). The global incidence of 11q23/MLL rearrangements ranges in different studies between 35 and 50% (4–8): the incidence by partners of 11q23/MLL rearrangements for t(9;11)(p22;q23), t(10;11)(p12;q23), t(11;19)(q23;p13.1), t(1;11)(q21;q23), and other translocation gene partners are approximately 10–20, 7–9, 1–4, 4–5, and 15%, respectively (4–8).

Translocation t(1;22)(p13;q13) can be found in a relevant proportion of infants (from 5 to 30%), their AML cytological variant mainly belonging to the FAB-M7 subtype. A complex karyotype (defined as three or more cytogenetic alterations, including one structural, and absence of favorable aberrations, or *MLL* rearrangements) seems to be another cytogenetic feature rather frequent in infants, with reported percentages of occurrence in the order of 14% under 2 years of age, this value decreasing to 4–7% in older children (4, 9). Conversely, younger children, particularly under 1 year of age, show a significantly lower frequency of cytogenetically normal (CN)-AML, compared with older children (4, 9).

Another distinctive trait of infant AML is the low frequency of favorable cytogenetic features, namely core-binding factor (CBF) abnormalities and t(15;17). In AML-BFM-98 and -2004 studies (4), no t(8;21) was observed in children under 2 years of age, while inv (12) or t(16;16) were found in only 4% of patients under 1 year in contrast with a frequency of 9% in older children (4). Acute promyelocytic leukemia is also extremely rare in infants, where it has a frequency of 1–2% compared with percentages of 6–7% in older children (4, 9).

The t(7;12)(q36;p13) and t(7;12)(q22;p13), often accompanied by trisomy of chromosome 19 or, in some cases, by trisomy 8, are also almost exclusive of this age group (13). The incidence of this latter rearrangement could be underestimated because of the inherent difficulty in detection through the use of conventional cytogenetics (14, 15).

Abnormalities of 12p, which characterize a cytogenetic group of patients with adverse outcome (16), were reported to occur with higher frequency in infants under 1 year of age (5 vs 1% and 0.3% in children aged 1–2 years and 2–10 years, respectively) (4).

### Molecular aberrations

The principal molecular aberrations of AML detected in children younger and older than 2 years of age are detailed in Figure 1B.

Mutations of the nucleophosmin 1 (*NPM1*) gene, primarily observed in patients with normal karyotypes and generally associated with a favorable prognosis (3, 17), are extremely rare in infants AML (8, 12, 18). The same consideration applies to the bi-allelic mutations of *CEBPA* (19). In the experience of the Children’s Oncology Group, among 193 infant patients analyzed, the *CEBPA* mutation was found in only 2% of cases, in contrast with 3 and 7% in the 3–10 years and over 10 years age group, respectively (19).

While, as mentioned above, *MLL*-gene rearrangements resulting from translocations are extremely frequent under the age of 1 year *MLL*, partial tandem duplications (PTDs) are rare in pediatric AML and generally not detected in infants (20).

The prevalence of *FLT3-ITD* mutation in childhood AML usually presents stepwise increase with age and thus its frequency in infant AML is significantly lower (2 and 5% of patients aged <1 year and 1 to <2 years, respectively) than in older children (8, 21). The point mutations of the activating loop of the FLT3 receptor (FLT3-TKD) seem to be slightly more prevalent in infants than *FLT3-ITD*, being detected in up to 5% of cases (21).

Regarding *RAS*-pathway aberrations, patients under and over 2 years of age show almost similar frequencies of *N-RAS* (15–20%) and *PTPN11* (2–3%) mutations, whereas *K-RAS* mutations seem to be more frequent in children over 2 years (9, 22). Overall, taking together all *RAS*-pathway mutations, their frequency in the age group under and over 2 years is similar (22 vs 21%, respectively) (9). Likewise, the frequency of activating mutations of the c-*KIT* receptor does not differ in infants and older children (5–10% in both groups) (9).

The recently described cryptic fusion transcript *CBFA2T3-GLIS2*, which is a recurrent feature of pediatric FAB-M7 and CN-AML, predicting poor outcome (11), is much more frequently detected (9.5%) in infants than in older patients (8).

## Prognostic Factors

Several studies have analyzed the clinical and biological factors influencing the outcome of infant AML, with conflicting results. A favorable cytogenetics and a blast count after induction therapy <5% have been shown to be the most powerful risk factor for the outcome of infant AML (4, 8).

Male gender and hyperleukocytosis were associated with poorer prognosis in children with AML aged 12 months or less in one study (23). Nevertheless, these data have not been confirmed in other studies (4, 7, 24). An analysis of 299 children with AML treated in four consecutive clinical trials between 1980 and 1997 (24) showed that FAB M4 or M5 was an independent prognostic factor predicting better outcome in children younger than 2 years. This finding was confirmed, although at a non-statistically significant level, in other experiences (7) (3-year event free survival – EFS of 80.8% reported by the Japanese group vs 56.1%, *P* = 0.105).

Central nervous system involvement at diagnosis does not influence the survival of infants with AML, although it is associated with a subsequent higher incidence of CNS relapse (2, 8, 24, 25).

In the majority of studies, the presence of *MLL* rearrangements did not affect outcome (4, 7, 8). One study (24) showed that t(9;11) confers a favorable outcome (5-years EFS 70 ± 16 vs 25% ± 7%, *P* = 0.01) to children with AML <2 years, but this finding was not confirmed in other trials (17, 26). Paucity of data do not permit to speculate on the prognostic role of complex or monosomal karyotype in infants, while an adverse prognostic effect is evident for older children (1, 27).

As already mentioned, a recently identified risk factor is the *CBFA2T3-GLIS2* fusion transcript. Indeed, the subgroup of infants harboring this abnormality have been reported to have a significantly worse prognosis as compared with *CBFA2T3-GLIS2-*negative infants (EFS 32.3 vs 59.6%, *P* < 0.05) (8).

## Treatment of Infant AML

In principle, management of young children with AML does not differ from that of older children. Neither the use of intensive chemotherapy nor the eligibility to hematopoietic stem cell transplantation (HSCT) are precluded by the immaturity of organs (lung, liver, brain) in children younger than 1 year. Differences in pharmacokinetic and pharmacodynamic profiles of certain drugs (e.g., cytarabine) could increase the susceptibility of infants to develop toxicities (see Section “Toxicity”), this observation translating on one hand into the need of adapting dosages of cytotoxic drugs and on the other into the opportunity that these peculiar patients be treated in centers with a recognized expertise. Similarly to what is employed in older children, an induction therapy combining antracycline and cytarabine (Ara-C) represents the backbone of initial treatment in use by many collaborative groups, while repeated courses of high-dose Ara-C in combination with other cytotoxic agents are largely used as conventional post-remission therapy. Allogeneic HSCT has been widely utilized to consolidate the state of remission also in infants, although the benefit deriving from this approach in comparison to repeated courses of chemotherapy has been questioned in recent years in view of a delicate balance between risk of relapse and late effects. CNS-directed therapy does not differ from that received by older children, being based on intrathecal cytarabine injections, with dose adjusted by age. Cranial radiation has been used (4), but never under 15 months of age due to the high risk of inducing permanent severe neurological sequels.

The outcome of infants with AML significantly improved over the last three decades, thanks to the intensification of therapy and the advances in risk assessment, together with the progress in supportive care (3): the EFS rate has increased from 30% in ‘80s–‘90s (24, 28) to about 50–60% in recent pediatric AML studies (4, 6, 8). The results of the most important AML trials involving infants are summarized in Table 1. Head-to-head comparison among all these results has to be made with caution, in view of different follow-ups (from 3 to 8 years), different selection of age groups (<1 year of age or 0–2 years of age), and possible exclusion of neonates, or of patients experiencing early death (ED).

**Table 1:** **Treatment outcome of children younger than 2 years in recent pediatric AML trials**.

Study	Years of study	N°	CR (%)	IF (%)	ED (%)	CIR (%)	OS (%)	EFS (%)	References
AIEOP LAM-92	1992–2001	39^b^	–	–	–	–	–	51	Pession et al. (29)
AIEOP-AML 2002/01	2002–2011	63^a^	82	14	3	31	74	55	Masetti et al. (8)
NOPHO-AML93	1993–2001	57^b^	–	–	–	–	–	54	Lie et al. (30)
MRC (AML 10, 12)	1994–2002	57^a^	89	0	11	25	65	59	Webb et al. (6)
ANLL91	1995–1998	35^a^	91	–	–	–	76	72	Kawasaki et al. (7)
BFM (AML 98, AML 2004)	1998–2010	125^a^	85	7.5	7.5	34	61 (AML 98)	44 (AML 98)	Creutzig et al. (4)
							75 (AML 2004)	51 (AML 2004)	
POG 8821	1988–1993	122^b^					33	22	Ravindranath et al. (31)
LAME 89/91	1988–1998	42^a^	85				82 (autograft)	37	Perel et al. (32)
							15 (Cht)		

*^a^Studies including children <1 year of age*.

*^b^Studies including children aged 0–2 years*.

*AIEOP, Associazione Italiana Ematologia e Oncologia Pediatrica; BFM, Berlin-Frankfurt-Munster; LAME, Leucamie Aique Myeloid Enfant; MRC, Medical Research Council; NOPHO, Nordic Society of Pediatric Hematology and Oncology; POG, Pediatric Oncology Group; Cht, chemotherapy; CIR, cumulative incidence of relapse; CR, complete remission; ED, early death; EFS, event free survival; IF, induction failure; OS, overall survival; y, years*.

### Treatment results by protocols

The most significant experiences (both for numbers of infants enrolled and observation time) of treatment analyzing separately the results of infants with AML are summarized below and reflect different approaches of post-remissional treatment.

Infants treated according the AIEOP AML 2002/01 Protocol (8) received two courses of induction therapy with idarubicin, Ara-C, and etoposide followed by two consolidation courses (high-dose Ara-C combined with etoposide and mitoxantrone during the first and second course respectively). The post-remissional treatment was largely based on autologus or allogeneic HSCT (33). The 8-year overall survival (OS) and EFS were 74 and 55%, respectively. The complete remission (CR), ED, induction failure rates, and cumulative incidence of relapse (82, 3, 14, and 31%, respectively) did not show any significant differences compared with those of older children.

A similar approach was reported in the MRC AML-10 and -12 trials (34) where infants achieving CR, except for good risk children, were eligible for matched family donor (MFD) HSCT. The main novelty of the AML-12 trial was a randomization between four and five courses of chemotherapy. A better outcome for younger children emerged from the comparison by age, the 5 years OS and EFS being 65 and 59% vs 56 and 47% in infants and in children aged 10–15 years, respectively (*P* = 0.02) (6). These findings were explained by a lower relapse rate in infants (*P* = 0.02) while the CR and ED rates were 89 and 11% in patients <1 year, similar to those observed in older children (*P* = 0.5, *P* = 0.06). Resistant disease resulted to be less frequent in younger patients, while the percentage of death during induction was reported to be higher in infants than in older patients (11 vs 4%, *P* = 0.06).

The 125 infants enrolled in the AML-BFM-98 (59 patients) and -2004 (66 patients) trials between 1998 and 2010 (4) (96% allocated in the HR group, according to their clinical and biological features and the response to therapy) received a standard induction therapy, followed by four courses of chemotherapy including high-dose Ara-C and anthracyclines. In the AML-BFM-2004 trial, patients were randomly assigned to receive liposomal daunorubicin in place of idarubicin in induction. The 5-year OS and EFS improved from 61 and 44% in the AML-BFM-98 to 75 and 51% in the -2004 trial, respectively (4). CR rate, OS, EFS, ED, and resistant disease of HR infants, and older HR patients were not statistically significant different, being 85, 66, 47, 7.5, and 7.5%, respectively.

The LAME French Cooperative Group (32) reported outcome of 42 treated according to studies LAME 89-91 where after an induction phase of mitoxantrone plus Ara-C, a consolidation based on high-dose Ara-C and asparaginase was given. As post consolidation therapy, 14 infants in CR1 underwent AUTO-HSCT. In all, 17 relapses and two deaths in CR occurred. The EFS was 37.3%. Disease free-survival (DFS) was 64.7% with autograft (*n* = 14) and 15% with chemotherapy alone (*n* = 18) (*P* = 0.12), and OS was, respectively, 82.7 vs 15.7% (*P* = 0.01). It is of interest that in this study, despite a satisfactory CR rate in infants (85%), the high relapse rate resulted in an extremely low OS for those treated with chemotherapy alone.

The Japanese collaborative group reported a 3-year OS and EFS of 76 and 72%, respectively, in infants with AML, mainly treated with intensive chemotherapy only (7). The 35 infants analyzed received an induction therapy including etoposide, Ara-C, and mitoxantrone, followed by four different courses of intensification therapy with etoposide, Ara-C, and anthracyclines, or vincristine. The outcome results of this study should be compared with caution to the others, because of the relatively small number of patients analyzed and the short follow-up.

### Role of HSCT for infant AML

The last decades have seen parallel improvements in chemotherapy-based and HSCT regimens for the treatment of infants AML. There is not a general consensus on indication for HSCT in CR1 for this category, being the use of HSCT mainly questioned in view of the potentially severe side effects, such as growth hormone deficiency, abnormal pubertal development, and hypothyroidism, correlated to the transplant procedure (35–38). In the absence of randomized studies comparing HSCT with other types of post-remission therapy in infants, it is extremely hard to define a clear indication on which infants would benefit of an allograft. The presence of a matched sibling donor as an HSCT indication, has, in most protocols, been replaced by risk assessments based upon disease and response-related factors.

Although the long-term toxicity associated with HSCT cannot be neglected, in view of the HR characteristics of almost all infants, transplantation holds the potential to still qualify as the treatment associated with the lowest risk of leukemia recurrence, in particular in subgroups of infants with worst prognosis (i.e., those with more than 5% blasts at the end of induction therapy or those carrying unfavorable cytogenetic/molecular lesions). The AIEOP experience, largely based on HSCT as consolidation shows how a good outcome, is not compromised by unacceptable TRM or high late effects rate. In the 2002/01 study (8) where 46 out of 63 children aged <1 year received HSCT in CR1, the risk of fatal events occurring after transplantation was extremely low (i.e., 1%). Since also only 10 out of the 46 infants relapsed, it is reasonable to speculate that HSCT significantly contributed to the favorable outcome of patients. This hypothesis is further corroborated by the observation that an allograft was able to guarantee continuous CR to 3 out of the 9 patients not responding to the induction treatment and to 5 out of the 10 patients relapsing after a first HSCT included in the study (8). As far as the incidence of side effects is concerned, among transplanted infants in the AIEOP study, 14% experienced growth deficiency, 3% decreased cardiac function, 9% hypothyroidism, and 6% developed impaired cognitive function.

Despite these favorable data, the role of allogeneic HSCT in CR1 of infants with AML is disputed. Indeed, the Japanese group reported good outcome in 35 infants, using allogeneic HSCT in CR1 only in 6 of them (7). Likewise, Creutzig and Colleagues reported an EFS comparable to that of the AIEOP group using ALLO HSCT in CR1 in only 14 out of the 125 infants reported in their more recent study (13 of them were alive and disease free) (4). They also prospectively evaluated the impact of matched sibling donor HSCT in children with AML in CR1 showing that the DFS for children younger than 2 years was not different (46% vs 53%, *P* = 0.53) (39). This said it is noteworthy to consider that children with 11q23 aberrations treated in the AML BFM98 study had a significantly better 5-year DFS when given allogeneic HSCT in CR1, the advantage offered by transplantation being even more evident when taking into account the 5-year OS (94 ± 6 vs 52 ± 7% of children treated with chemotherapy only, *P* = 0.01) (39). Moreover, in a recent retrospective analysis conducted by Eurocord/EBMT on 95 infants with AML receiving single-unit cord blood transplantation after a myeloablative preparation, the reported 4-year DFS was 82% for those infants who were transplanted in CR1 (40).

The issue whether HSCT should be largely employed in infants with AML in CR1 or rather mainly reserved to relapsing patients remains unsolved and needs to be addressed in future studies.

## Toxicity

Regarding therapy-related toxicity in infant AML, it has been severely reduced in the last decades due to the advances in the supportive care. The increased susceptibility to toxicities of infants patients results from immaturity of lung, liver, and brain function, as well as a distinctive pharmacokinetics and pharmacodynamics profile of drugs such as Ara-C (2, 4, 5). These findings have led to the adjustment of drug dosage on the basis of body weight (mg/Kg) instead of body surface. High-dose Ara-C was age-adjusted in children younger than 2 years in some trials because of reduced clearance of this drug (4, 27, 41).

The toxicities observed in the Japanese study (7) included 9 septic episodes (4 during induction and 5 during intensification therapy), 2 cutaneous abscess, 2 viral pneumonia, 1 neurotoxicity, 1 hemorrhagic cystitis, and 1 hypocalcemia. The majority of the patients developed febrile episodes associated with myelosuppression.

Regarding gastrointestinal toxicity, Webb et al. did not observe (6) age-related incidence of oral toxicity (*P* = 0.8) and nausea or vomiting (*P* = 0.01). On the contrary, the study revealed a higher prevalence of severe diarrhea in younger children, the incidence decreasing from 38% in infants to 16% in patients aged 10–15 years (*P* = 0.002).

In the BFM studies (4), infants developed significantly higher rates of pulmonary problems (34% in infants, 24% in children aged 1–2 years, 14% in older patients, *P* = 0.01) and compromised general condition (50%, 43%, 31% in infants, children aged 1–2 years and older patients, respectively) after induction. Moreover severe infections occurred in 42% of infants compared to 29% of children aged 2–10 years (*P* = 0.08). Severe mucositis occurred less frequently in infants (4, 8). No differences in late effects were observed between infants who did or did not receive HSCT (59 vs 43%, *P* = 0.39) (8), but these data need to be confirmed after a longer follow-up.

## Conclusion

In summary, infants with AML represent a cohort of patients with peculiar clinical and biological features. The presence of remarkable differences in terms of molecular lesions or clinical characteristics of infant AML have a limited impact on their outcome. This latter, after both frontline and relapse treatment, has improved considerably over the last 10–15 years, being now super imposable to that of older children in the experiences of the major collaborative groups. Intensive AML treatment is feasible in this young subgroup, and toxicities are manageable. The effort of future trials should address defining which subgroups of infants, considering cytogenetic/molecular features and response to treatment, require more aggressive therapy including HSCT.

## Conflict of Interest Statement

The authors declare that the research was conducted in the absence of any commercial or financial relationships that could be construed as a potential conflict of interest.
